# Exosome-Mediated Signaling in Epithelial to Mesenchymal Transition and Tumor Progression

**DOI:** 10.3390/jcm8010026

**Published:** 2018-12-27

**Authors:** Alice Conigliaro, Carla Cicchini

**Affiliations:** 1 Dipartimento di Biopatologia e Biotecnologie Mediche, University of Palermo, 90100 Palermo, Italy; 2 Dipartimento di Medicina Molecolare, Sapienza University of Rome, 00161 Rome, Italy

**Keywords:** tumor niche, Epithelial–Mesenchymal plasticity, cancer-derived exosomes, extracellular vesicles, metastasis

## Abstract

Growing evidence points to exosomes as key mediators of cell–cell communication, by transferring their specific cargo (e.g., proteins, lipids, DNA and RNA molecules) from producing to receiving cells. In cancer, the regulation of the exosome-mediated intercellular communication may be reshaped, inducing relevant changes in gene expression of recipient cells in addition to microenvironment alterations. Notably, exosomes may deliver signals able to induce the transdifferentiation process known as Epithelial-to-Mesenchymal Transition (EMT). In this review, we summarize recent findings on the role of exosomes in tumor progression and EMT, highlighting current knowledge on exosome-mediated intercellular communication in tumor-niche establishment, migration, invasion, and metastasis processes. This body of evidence suggests the relevance of taking into account exosome-mediated signaling and its multifaceted aspects to develop innovative anti-tumoral therapeutic approaches.

## 1. Introduction

The lipid-bilayer extracellular vesicles (EVs) include at least three main classes of vesicles that differ in dimension, biogenesis and biophysical properties: exosomes, microvesicles, and apoptotic bodies. Here, we focused on exosomes, small vesicles with an average diameter from 30 to 200 nm that originate inside the Multi Vesicular Bodies (MVBs) and are released by the parental cell after MVB fusion with plasma membrane.

Although exosomes were first identified as garbage disposal [1,2], current knowledge highlights the direct role of these vesicles in governing physiological and pathological conditions by transferring information from producing to receiving cells. Exosomes, indeed, may signal in autocrine but, most importantly, in paracrine and endocrine manner, being taken up by neighboring cells or carried to distant sites. Thus, they assure the horizontal transfer of specific bioactive molecules (including proteins, lipids, RNAs, and DNA [3]) from donor to recipient cells.

Exosomes are ordinarily released by different cell types [4]. They have been identified in various body fluids (including semen, blood, urine, cerebrospinal fluid, and milk) and their role is strongly associated with the cytotype of the producing cells. For example, exosomes participating in supporting immune response [5] and, as vesicles secreted by cells of the nervous system, have been found to coordinate myelin membrane biogenesis, neuronal development, transmission and regeneration [6,7,8]. Interestingly, several studies recently reviewed by Guay and Reguazzi [9], pointed to the involvement of exosomes in a “new endocrinology”, being mediators of the crosstalk between metabolic organs.

Despite the exosome role in homeostasis maintenance and physiology, the most recent intensive investigation was focused on the involvement of these EVs in pathological processes. Particularly in cancer, the regulation of the exosome-mediated intercellular communication may be reshaped. Exosomes, indeed, may carry messages from transformed to healthy cells or to other cells in the tumor or they may signal in an autocrine manner back to the producing tumor cell, thus allowing relevant changes in recipient cells behavior and microenvironment alterations. Notably, exosomes may deliver signals able to induce an Epithelial-to-Mesenchymal Transition (EMT), a transdifferentiation process that underlines tumor dissemination.

In this review, we focus our interest on the role of exosomes during the EMT process in tumor progression. Starting with an overview on the molecular composition of these vesicles, with a focus on their emerging heterogeneity, we highlight current knowledge on the exosome-mediated intercellular communication in tumor niche establishment, migration, invasion, and metastasis processes.

This body of evidence suggests the relevance of taking into account exosome-mediated signaling and its multifaceted aspects to develop innovative anti-tumoral therapeutic approaches.

## 2. Exosome Heterogeneity and Cargo Composition

Exosomes are generally characterized by markers such as tetraspanins (e.g., CD63 [10]) and heat shock proteins (HSP70 and HSP90 [11]) while their biogenesis involves the endosomal sorting complex required for transport (ESCRT; [12]), Rab proteins [13], syndecan-syntenin-Alix [14], and others. Despite that, the improvement in the technologies adopted for exosomes isolation and characterization highlighted that, even if originating within the MVBs and presenting common markers, exosomes may show physical and chemical differences. Therefore, for a better comprehension of the variety and the apparent discrepancy of current literature reports, we should consider that exosomes may exhibit per se heterogeneity, both in physiological and in pathological conditions.

With respect to exosome features, Kowal and collaborators [15] demonstrated by a quantitative proteomic analysis, that high-speed ultracentrifugation, considered the gold-standard purification method for exosomes, allows the isolation of four different populations, among which, only two are associated with the endosomal pathway, and can be further separated for the different expression/enrichment in tetraspanin Cluster of Differentiation (CD) 63, CD81, and/or CD9. Interestingly, Willms et al. [16] demonstrated that several cell types release two major subpopulations of exosomes with distinct molecular compositions and biological properties. At least two different types of exosomes have been recovered from saliva, differing in size and content in term of proteins and RNAs [17,18]. Furthermore, the Lotvall group proved that human mast cells release two distinct exosome families that, separated by floatation on a density gradient, present “substantial” differences in RNA species content as demonstrated by microarray and Next Generation Sequencing (NGS). Interestingly, while RNA from Low Density exosome correlated with cellular mRNA, High Density exosomes were enriched in non-coding RNA (ncRNA). Moreover, differences in RNA signatures and protein patterns led the authors to hypothesize about possible different exosome biogenesis pathways [19].

Exosomes with different protein composition and surface markers have been identified after flotation onto sucrose gradient by Bobrie et al. [20]. These authors reported that high-speed ultracentrifugation co-purified vesicles bearing the endosomal tetraspanin CD63 together with smaller vesicles which exposed the CD9 tetraspanin and the peripheral membrane-associated protein Mfge8. Most interestingly, the discovery that Rab27a, a small GTPase known to be involved in exosome secretion, is required for the release of the only CD63 positive exosomes, enforcing the hypothesis that heterogeneity comes from different molecular mechanisms of formation and secretion of exosomes.

With respect to cargo molecules, exosomes embed several macromolecules e.g., lipids, metabolites, nucleic acids and proteins (the complete lists of exosome embedded macromolecules can be found on ExoCarta [21] or Vesiclepedia [22]. Cargo molecules depend on the cell of origin, the change in response to physiological and pathological conditions [23] and maintain their biological function when transferred to the receiving cells, impacting their fate [24]. Nucleic acid analysis revealed, inside exosomes, an abundance of RNA families that, protected from RNases by lipoprotein envelop, maintain their functions. Notably, specific subsets of miRNAs appear to preferentially localize to exosomes [25,26,27] and, as demonstrated by Pegtel at al. [28], exosome-mediated miRNAs delivery directly modulates specific targets once in the cytoplasm of receiving cells. Even if numerically less abundant than small RNAs, long non-coding RNAs (lncRNAs) have been found in exosomes released by different cell types, specifically by tumor cells, thus representing new specific tumor markers [29,30,31]. However, further studies are required to fully understand the effects induced by non-coding RNAs in target cells, specifically concerning lncRNAs, whose pleiotropic roles make them protagonists in the control of gene expression from epigenetics to miRNAs inhibition. Concerning this, two interesting manuscripts from the Lorico group [32,33] demonstrated that internalized exosomes, or part of these, can directly reach the nucleus of receiving cells. These data initiate interest on all of the compounds that, transported by the exosomes, have a nuclear biological activity (e.g., transcription factors, histone modification enzymes, and lncRNAs).

Deeper investigations are required also to characterize the loading of specific macromolecules. The study of miRNA-motifs mediated loading are so far limited to a few reports that identified specific RNA-binding-proteins and some miRNA consensus sequences mediating the process [34,35,36]. While the mechanisms of selective loading of RNAs, as well as proteins, in exosomes are still poorly understood, it is conceivable that pH [37] and hypoxia [38] may affect both the entity of the release and the sorting of a specific content. The lack of standardized well-characterized methods to isolate, purify, and quantify the exosomes further limits the study of their content as cell signature.

The high variability of exosome-induced effects is also determined by the type of interactions occurring between exosomes and target cells that, as recently reviewed in [39], are governed by numerous factors. Depending on their origin, exosomes have been found to interact preferentially with specific cell types, and this interaction seems to be strongly conditioned by the integrins exposed on the exosome surface [40]. It is conceivable that an extensive proteomic analysis of adhesion proteins, such as extracellular matrix proteins (e.g., fibronectin and laminin) and tetraspanins might help to predict exosome-cell interactions but we lack efficient protocols for the isolation of outer membrane proteins only.

Once in touch with target cells, the strategy used to transform these are multiple. First, exosomes may activate receiving cells from the outside, through a ligand/receptor interaction and subsequent activation of downstream pathways.

Paradigmatic is the immune tolerance induced by several cancer cells through exosomes, which express death signals as the PD-L1 (programmed death-ligand 1) or Fas Ligand, and systemically induce apoptosis in receiving T cells and Natural Killer (NK) cells [41,42]. Ligand /receptor interaction have a role also in exosome migration around the body as recently demonstrated for CCR7 (CC-chemokine receptor 7) that, exposed on the Dendritic Cell exosomes, contributes to both their migration on spleen and the induction of inflammation [43]. Exosomes expressing the amphiregulin (AREG), isolated from several tumor cells, have been found able to activate the Epidermal Growth Factor Receptor (EGFR) in receiving cells thus affecting the bone marrow microenvironment [44] or promoting bone metastases [45]. Finally, several groups demonstrated a regulation of the Transforming Growth Factor (TGF)β pathway mediated by membrane-bound molecules [46,47] or (GPI)-anchored cell surface glycoprotein [48].

In most cases, the interaction with cellular receptors drives exosome internalization. Receptors or proteins located on the EV surface participate in fusion, endocytosis or phagocytosis with subsequent release of the exosome content in the receiving cells.

## 3. EMT Associated with Tumor Progression: The Role of Exosomes

EMT is a physiological or pathological transdifferentiation process in which epithelial cells lose their cell-cell contacts and apicobasal polarity and acquire mesenchymal properties, coupled to the ability to migrate and to invade the surrounding tissues. EMT is crucial in organogenesis, development, wound healing and regeneration but it is aberrantly activated in tumor progression and metastasis (for review, [49]). EMT, indeed, allows in situ differentiated cells to acquire the ability to migrate out of the primary tumor, invading basement membrane and entering the vasculature. Transitional tumor cells exit from circulation and migrate into the tissue parenchyma in potentially secondary tumor sites. In this process of colonization of target tissues by metastatic cells (as well as during morphogenesis), the shift toward a mesenchymal state is often reversed by an inverse Mesenchymal-to-Epithelial Transition (MET). The MET occurs in different microenvironments and it is necessary to support the reacquisition of epithelial features to seed metastasis [50,51].

EMT/MET plasticity implies a profound reprogramming of gene expression mainly orchestrated by specific “master” transcription factors, known as EMT-inducing transcription factors (EMT-TFs; i.e., ZEB1; SIP1/ZEB2; Twist1; Twist2; E12/E47; Tbx3; the Snail family members Snail2 (Slug), Snail3 (Smuc) and, in particular, Snail1 (Snail) [52,53,54,55,56]. The EMT-TFs primarily act as repressors of the epithelial genes and may guide the recruitment of the epigenetic machinery to the chromatin context, thus allowing the proper regulation of gene expression [57,58].

Rather than a simple shift between two alternative states (i.e., the mesenchymal and the epithelial phenotype), the current view is that the EMT/MET implies multiple and dynamic transdifferentiation states. This greater flexibility may result in a “partial EMT” or in the co-presence of epithelial and mesenchymal traits, as in the hybrid “metastable” features identified in several tumors [59,60,61] and attributed to a stem phenotype [62,63,64,65]. The complexity of the EMT/MET phenotypes reflects the complexity of the regulatory circuitries that, beyond the transcriptional control, also involve several ncRNAs, including miRNAs (e.g., miR-200 family or miR-34) [66,67,68] or lncRNAs (e.g., HOTAIR) [57]. Notably, transitional tumor cells need to be continuously reprogrammed to adapt to different microenvironments and to ensure tumor growth and metastasis [69,70,71].

Exosome composition profoundly differs between untransformed and transformed cells [72,73,74] and increasing evidence suggests that tumor-derived exosomes (TDEs), as well as exosomes from tumor associated cells in the microenvironment (TME), exert a key role in the regulation of tumor growth and survival as well as tumor invasion, angiogenesis, and metastasis.

**Figure 1:**
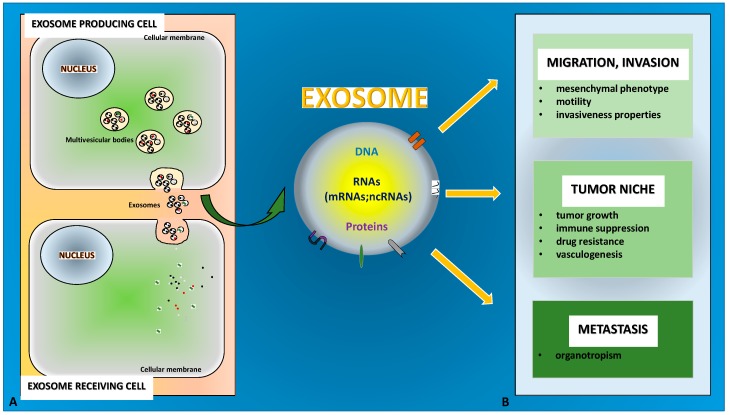
The role of exosomes in Epithelial-to-Mesenchymal Transition (EMT) and tumor progression is depicted. (**A**) Exosomes originate from the multivesicular bodies that release them by fusing with the cellular membrane. (**B**) Exosome cargo content of DNA, RNA (including ncRNA) and proteins specifically mediates cell–cell communication in EMT and in the associated tumor progression to promote different outcomes.

Notably, TDEs may carry pro-EMT cargoes that include EMT inducer molecules, e.g., TGF-β, Hypoxia-inducible factor (HIF)1α, β-catenin or miRNAs, such as miR-23a. All this content is able to (i) confer mesenchymal properties to epithelial cells, (ii) promote the initiation phase of the epithelial tumor metastasis (when in situ tumor cells migrate out of the primary tumor, invading basement membrane and entering the vasculature) and (iii) guarantee tumor-microenvironment cross-talk [75,76,77,78,79,80] (Figure 1A,B).

It is conceivable that a fine tuning of the EMT plasticity may result in the capacity by the cell to export specific bioactive molecules and, vice versa, the exosome-mediated signaling may impact on the EMT/MET dynamics. Coherently, exosomes from transitional cells exert a role in the regulation of tumor niche, migration, invasion, and metastasis.

### 3.1. Exosomes in Tumor Niche

In primary tumor, exosomes contribute to the definition of tumor niche by promoting tumor growth (despite nutrient deprivation and stress condition), immune suppression and drug resistance, and enhancing vasculogenesis.

Cell growth can be stimulated by cytokines that, loaded in TDEs, are then transported to closer tumor cells; Raimondo et al. demonstrated that exosomes released by chronic myeloid leukemia cells promoted tumor cell growth and inhibited apoptosis by activating TGFβ receptor [46]. Moreover, TDEs can induce proliferation of adjacent cells by non-coding RNA-mediated signaling e.g., the miR-27 in gastric cancer exosomes [81] and the lncRNA c-Myc-Upregulated (MYU) in prostate cancer exosomes [82]. Meanwhile, paracrine stimulation of tumor cell proliferation is induced by cancer associate fibroblasts (CAFs). These cells are pivotal players in the tumor microenvironment by stimulating Cancer Stem Cells maintenance and EMT [83]. Interestingly, exosomes released by tumor cells may activate resident fibroblasts inducing CAFs [81,84]. In bladder cancer, Ringuette et al. showed that CAF activation is mainly due to the induction of TGFβ/Small Mothers Against Decapentaplegic (SMAD) pathway resulting from the transport of TGFβ by TDEs [84]. Furthermore, in gastric cancer an important contribute to fibroblasts activation is due to the exosome mediated transport of miR-27a [81].

The contribution of CAF released exosomes to tumor growth is supported by studies investigating CAF proteome and transcriptome that identified a huge number of molecules with pro tumorigenic activity [85,86]. Interestingly, Zhang and colleagues underlined the possibility that exosomes may carry both pro- and anti-tumor factors. These authors, in fact, by comparing miRNA sequencing of exosomes derived from CAFs and exosomes secreted by fibroblasts from HCC (Hepatocellular Carcinoma) patients, demonstrated that CAF-derived exosomes stimulate cell proliferation lacking protective elements such as the miR-320a that, on the contrary, is transported by NF-exo and is able to inhibit HCC growth through MAPK targeting [87].

TDEs may also contribute to acidification of the tumor microenvironment by modulating stromal cell metabolism. Recently, it was demonstrated that human melanoma-derived exosomes, once internalized by dermal fibroblasts, promote aerobic glycolysis and downregulate oxidative phosphorylation [88]. Meanwhile, the Nagrath group elegantly demonstrated that exosomes from CAFs downregulate mitochondrial activity and increase glycolysis. Furthermore, intra-exosome metabolomic analyses showed that exosomes contain metabolites, amino acids, and lipids, “which can fuel the metabolic activity of the recipient cells” [89].

Finally, as mentioned above, exosomes indirectly support tumor growth by favoring immune escape in different ways; they inactivate T cells or induce their apoptosis by cell surface interaction or after internalization [90,91,92]. With respect to immunomodulatory properties, TDEs exposing CD39 and CD37 on their surface can mediate T-cell suppression by extracellular adenosine production [93]. Meanwhile it was shown that the internalization by Kupffer cells of pancreatic ductal adenocarcinoma TDEs induced fibronectin production, thus promoting the gathering of bone marrow-derived macrophages and neutrophils and leading to liver pre-metastatic niche formation [94].

A key component of tumor microenvironment is the vascular network that supports tumor growth; several extracellular mechanisms take part in endothelial cell stimulation, among these TDEs contribute to modulating both angiogenesis and vascular permeability [95].

It is of note that the ability to promote angiogenesis has been mainly attributed to exosomes isolated from tumor initiating cells (TICs). In renal cell carcinoma and hepatocellular carcinoma for example, only CD105+ and CD90+ cells respectively, release exosomes able to stimulate production and release of the Vascular Endothelial Growth Factor (VEGF); TIC derived exosomes, once engulfed in endothelial cells, activate the VEGF autocrine loop through the delivery of different ncRNAs [31,96]. Recently, Sun et al. demonstrated pro-angiogenetic activity also in exosomes from Glioma stem cells, that stimulate endothelial cell motility by activating a miR-21/VEGF/Vascular Endothelial Growth Factor Receptor (VEGFR) 2 signal pathway [97]. In addition, exosomes can directly stimulate endothelial cell VEGF receptor through the delivery of 90-kDa VEGF (VEGF90k), which was found to interact with Hsp90 in extracellular vesicles [98].

Several observations correlate hypoxia, exosomes, and neo angiogenesis stimulation. Low oxygen partial pressure, a common characteristic of all types of cancer, induces activation and nuclear translocation of the transcription factor HIF1 that, inside the nucleus, interacts with several co-factors to induce the up regulation of a huge number of genes whose coordinated expression drives tumor cells to a most aggressive phenotype [99]. Among the HIF target is the VEGF. Evidence collected in past years demonstrated that hypoxia stimulates production and release of exosomes [100] that, in turn participate in promotion of tumor neo-angiogenesis as described by ref. [101] and enclosed references.

Although VEGF signaling is the best-validated pathway in angiogenesis, the refractoriness to anti-VEGF therapies in several cancers highlighted the involvement of VEGF-independent strategies in promoting tumor angiogenesis [102]. An interesting study performed by Tang et al. [103] demonstrated that exosomes, released by ovarian cancer cells, participate in cleavage and delivery of soluble E-cadherin that once delivered on endothelial cell surface, interacts with VE-cadherin and induces activation of β-catenin and Nuclear Factor kappa-light-chain-enhancer of activated B cells (NF-κB) signaling, resulting in endothelial cell migration, and tube formation in vitro and in vivo.

### 3.2. Exosomes in Migration, Invasion, and Metastasis

A body of evidence points to the role of exosome-triggered EMT in the inception of high metastatic potential that correlates with high motility and increased invasiveness. Exosomes from cancer cells were found to be able to activate intracellular pathways by transporting specific proteins such as phosphorylated tyrosine kinases receptor (RTKs) [104]. Exosomes from muscle-invasive bladder cancer induced a decrease in E-cadherin expression and enhanced migration and invasion of uroepithelial cells [79]. Similarly, exosomes from highly metastatic lung cancer cells induced an EMT associated with migration, invasion, and proliferation in recipient human bronchial epithelial cells [105]. Furthermore, exosomes secreted by highly metastatic MHCC97H hepatocarcinoma (HCC) cells conferred the ability to migrate and give invasiveness properties to low metastatic HCC cells by inducing EMT via MAPK/Extracellular signal-Regulated Kinase (ERK) signaling pathway activation [106]. Finally, human breast and colorectal cancer cells released full-length, signaling-competent EGFR ligands, i.e., amphiregulin, able to increase the invasiveness of recipient cancer cells [107].

Harris et al. investigated the role of exosomes released from different breast cancer cells, modeling different stages of metastasis. They showed that tumor cells of increasing metastatic potential are able to secrete exosomes with protein signatures different in identity and abundance; these exosomes increased cell migration proportionally, with exosomes from high-metastatic potential cells able to induce the greatest degree of cell movement [108]. Interestingly, xenograft tumor cell motility studies in the chorioallantoic membrane (CAM) of chick embryos revealed a key role of exosome secretion for the directional migration of fibrosarcoma cells. These vesicles, indeed, carry extracellular matrix (ECM) molecules promoting adhesion assembly [109].

Furthermore, Schillaci et al. [110] recently demonstrated that exosomes released by metastatic colon cancer cell lines affected tumor behavior promoting a more aggressive phenotype. They found that metastatic amoeboid cells (SW620) release exosomes that are enriched in Thrombin, activating RhoA/Rho-associated protein kinase (ROCK) pathway in receiving cells. This activation induces migration and invasion in primary tumor cells while, in endothelial cells, causes VE-cadherin delocalization and junction disruption.

Concerning exosomal lncRNAs, a role for BCAR4 (breast cancer anti-estrogen resistance 4) has been suggested in colorectal cancer development [111] while HOTAIR (Hox antisense intergenic RNA) was found overexpressed in bladder cancer patients and correlated with the invasiveness of the tumor [30]. Notably, HOTAIR has a key functional role in promoting EMT in different cell types [57,112,113].

With respect to TMEs, Luga et al. demonstrated that CD81-positive CAF-released exosomes induced in breast cancer cells the release of Wnt11 that, in an autocrine manner, promoted the activation of Planar Cell Polarity (PCP) [114]. Meanwhile, Condorelli’s group attributed the induction of EMT in breast cancer to three different miRNAs (miRs-21, -378e, and -143), delivered by CAF-derived exosomes [115]. These observations enforced the idea that cellular transformation, induced by exosome uptake, must be mediated by multiple biological compounds that converge on the same molecular pathways. Li and collaborators demonstrated that ovarian CAF-derived exosomes were enriched in TGFβ1 that may induce an EMT and an aggressive phenotype in ovarian cancer cells lines [116]. More recently, Zhao and colleagues demonstrated that human umbilical cord mesenchymal stem cells-derived conditioned medium was able to induce migration and invasion capability in A549 lung cancer cells by activating TGFβ-related pathways [117].

The ability to migrate and invade surrounding tissues can be enhanced by hypoxia-induced exosomes by shuttling different molecules. Firstly, exosomes released in hypoxia may contain hypoxia-inducible factors (HIFs), able to trigger EMT in recipient cells [78]. Coherently, Ramteke and colleagues showed that hypoxia-induced exosomes increased the invasiveness of prostate cancer cells by promoting the loss of E-cadherin [118]. Furthermore, exosome-mediated mechanisms to promote migration and invasiveness by tumor cells in hypoxia may involve lncRNAs, such as UCA1 in bladder cancer cells [119] or the lncRNA-regulator of reprogramming (RoR) in HCC [120], as well as specific miRNAs, e.g., miR-21 in oral squamous cell carcinoma [94] or miR-23a in lung cancer [121].

Interestingly, Zhou and colleagues reported that exosomes secreted by cervical squamous cell carcinoma (CSCC) cells may shuttle miR-221-3p, targeting vasohibin1 (VASH1), to human lymphatic endothelial cells (HLECs). This promotes migration in vitro as well as lymphangiogenesis and lymph node metastasis in vivo [122].

The role of TDEs is not limited to the primary tumor site and their ability to cross long distances within the body makes exosomes a suitable vehicle to trace the way of tumor metastases. TDEs promote the organotropism of metastatic tumors and contribute to pre-metastatic niche formation by showing “avidity” for specific recipient cells [11,40,94]. Notably, Hoshino and colleagues showed that the exosomal integrins guide the exosomes to specific secondary sites. Furthermore, exosomes from lung-, liver- and brain-tropic tumor cells preferentially fuse with lung fibroblasts and epithelial cells, liver Kupffer cells, and brain endothelial cells [40].

## 4. Conclusions and Perspectives

EMT exerts a key role in tumor progression and exosomes, released by transitional cells, transport specific signaling molecules to promote invasion, migration, metastasis, and microenvironment changes, able to sustain tumor growth and dissemination. In fact, the horizontally transferred TDE specific content promotes the acquisition by tumor cells of mesenchymal markers and increases cell motility, associated with a more aggressive phenotype. Furthermore, exosome cargo impacts on tumor niche establishment and regulates the tropism of metastasis (Table 1 and Table 2). Therefore, the identification of molecules (mRNA, ncRNAs, proteins) specifically enriched in exosomes from different tumor stages may represent an efficient real-time staging of tumor evolution or response to therapy, also in patients differing in gender or age. Notably, EVs may be isolated from body fluids, and several RNA and protein molecules have already been identified as potential diagnostic and prognostic biomarkers of different tumor types or different stages of the same tumor. Interestingly, the study of the secretome of HCC cells overexpressing the master transcriptional factor Slug, and exhibiting a partial EMT phenotype, showed the enrichment in exosomes of Fibronectin 1 (FN1), collagen type II alpha 1 (COL2A1), and fibrinogen gamma chain (FGG); therefore, these proteins may represent useful and non-invasive biomarkers associated with partial transitional cells [123]. Notably, a partial EMT may characterize circulating tumor cells (CTCs) that pose a metastatic risk for patients [124].

All further efforts in the study of biomarkers would have a great impact particularly on early diagnosis, considering that the development of non-invasive diagnostic tools currently represents a major challenge. Moreover, an optimization of exosome isolation protocols is required to better disclose the functional role of specific exosome cargo molecules. The isolation of exosomes with high purity and quality is still difficult and the demonstrated heterogeneity of exosomes further impairs the isolation efficiency; all these aspects represent a limitation, particularly for the study of low copy number molecule species, such as lncRNAs.

A deep understanding of mechanisms controlling the loading of specific molecules in exosomes is also needed. This field of study is to be considered still at infancy even if some mechanisms of sequence-specific miRNA sorting (by EXO- and hEXO-motifs) and specific heterogeneous nuclear ribonucleoproteins (hnRNPA2B1 and hnRNPQ) involved in the recognition of these signals, have been recently identified [34,35]. Interestingly, the sorting of lncRNA ARSR in exosomes by renal cancer cells also involves hnRNPA2B1 [128]; furthermore, a “zipcode” in the mRNAs may control their selective loading [129]. All of this evidence suggests common regulative mechanisms, at least for RNA loading, and opens the way towards possible innovative therapeutic strategies oriented to the selective modulation of RNA exosomal cargo by engineering signaling sequences. Other approaches could aim to interfere with, or promote, the in vivo sorting of these vesicles. The use of specific inhibitors, targeting key regulators of both exosome biogenesis and release, could be a suitable approach. For example, the release of EVs by primary hepatocytes and Huh7 cells may be reduced by inactivating mediators of the DR5 signaling pathway or ROCK1 inhibition. Interestingly, the ROCK1 inhibitor *fasudil* reduced serum levels of EVs in nonalcoholic steatohepatitis (NASH) mice and this reduction was associated with decreased liver injury, inflammation, and fibrosis [130]. The drug GW4869 was efficiently used to inhibit exosome biogenesis by interfering with sphingomyelinase function [125,126]. The release of exosomes was shown to be influenced by calcium and the monensin drug was found able to affect exosome biogenesis [131]. Further studies are still necessary to investigate the effective translational application of these protocols.

Remarkably, exosomes show low immunogenicity, high biocompatibility, and high efficacy of delivery. Considering all these features, they might be engineered to convey molecules of interest and achieve targeted therapeutic intervention. For a recent example, paclitaxel-loaded exosomes, modified to improve circulation time, were shown to selectively deliver the drug to target cancer cells and increase the survival rate of lung cancer patients [132].

Finally, an in-depth understanding of exosome cargo composition and functional role in EMT associated with tumor progression would pave the way for innovative therapeutic opportunities. In particular, further studies must be focused on the characterization and potential engineering in vivo and/or ex vivo of exosomes from EMT cells. The plasticity of transitional cells might, indeed, imply a fine-tuning regulation of the loading machinery that, in turn, might be mirrored by the great complexity of exosome cargoes.

## Figures and Tables

**Table 1:** Summary of recent evidence on exosome signaling molecules and their effects on tumor progression.

Exosome-Mediated Effect	Producing Cell	Specific TDE Content and Mechanism of Action	Reference
*Tumor cell proliferation*	Prostate cancer cells	lncRNAc-Myc Upregulated (MYU)-mediated upregulation of c-Myc by competitively binding miR-184	[82]
	Hypoxic bladder cancer cells	lncRNA-UCA (unknown mechanism)	[119]
	Hypoxic hepatocellular carcinoma cells	lncRoR-induced hypoxic responses (by downregulation of miR-145 and upregulation of Hypoxia-inducible factor 1 (HIF1)	[120]
	CAF from Human Oral Tongue Squamous Cell Carcinoma	MFAP5 (Microfibril Associated Protein 5)-induced activation of mitogen-activated protein kinase (MAPK) and AKT	[85]
	CAF from Hepatocellular carcinoma	MAPK activation by negative regulation of miR-320a	[87]
	CAF from pancreatic ductal adenocarcinomas	Snail and microRNA-146a upregulation	[125]
*EMT and metastasis of tumor cells*	Bladder cancer cells Colon cancer cell lines	RhoA/ROCK (Rho-associated protein kinase) signaling pathway activation and acquisition of migratory capacity	[79,110]
	Epstein-Barr-Virus EBV infected Nasopharyngeal carcinoma (NPC)	HIF1 upregulation	[78]
	metastatic melanoma cells	MET induced pro-vasculogenic and metastatic effects	[11]
	Hypoxic cancer cells	Activation of Epithelial-to-Mesenchymal Transition (EMT) genes in receiving cells	[101] and enclosed references
	Lung cancer cells	vimentin	[105]
	Hepatocellular carcinoma cells	MAPK/ERK (Extracellular signal-Regulated Kinase) signalling activation (unknown mechanism)	[106]
	High aggressive breast cancer	proteins involved in metastasis and invasion	[108]
	Prostate cancer cells	metalloproteinases induction and targeting of adherens junction proteins	[118]
	Hypoxic bladder cancer	lncRNA-UCA1 (Urothelial Cancer Associated 1) (mechanism of action unspecified)	[119]
	Metastatic breast cancer	miR-10b targeting HOXD10 (HomeoboxD10)	[126]
	CAF from Human Oral Tongue Squamous Cell Carcinoma	MFAP5 activation of MAPK and AKT	[85]
	CAF from Hepatocellular carcinoma	MAPK activation	[87]
	CAF from breast cancer	EMT activation by miRs -21, miR-378 and miR-143	[115]
	CAF from ovarian cancer	TGF (Transforming Growth Factor) β1-induced EMT	[116]
	Mesenchymal stem cells	TGFβ1 activation of Smad2/3, Akt/GSK (Glycogen synthase kinase)-3β/β-catenin, NF-κB (Nuclear Factor kappa-light-chain-enhancer of activated B cells), ERK (Extracellular signal-Regulated Kinase), JNK (c-Jun N-terminal kinase and p38 MAPK (mitogen-activated protein kinase)	[117]

**Table 2:** Summary of recent evidence on exosome signaling molecules and their effects in the tumor microenvironment.

Tumor Microenvironment Modification	Producing Cell	Specific Content and Mechanism of Action	Reference
*CAF activation*	Gastric cancer cells	miR-27a-mediated downregulation of CSRP2 (cysteine and glycine rich protein 2)	[81]
	Bladder cancer cells	TGFβ–induced SMAD (small mothers against decapentaplegic) activation	[84]
	Pancreatic ductal adenocarcinomas	Stellate cells activation and induction of a pro-inflammatory milieu. (unknown mechanism)	[94]
	Prostate cancer cells	Induction of TGF-β2, TNF1α (Tumor necrosis factor1 α), IL6 (Interleukin 6), TSG101 (Tumor susceptibility gene 101), Akt, ILK1 (Integrin-linked kinase1) and β-catenin.	[118]
*Angiogenesis and vascular permeability*	Cancer Stem Cells from Hepatocellular Carcinoma	lncRNA H19-mediated VEGF (Vascular endothelial growth factor) induction	[31]
	Metastatic breast cancer	miR-105 targeting of ZO-1	[95]
	Glioma stem cells	miR21-mediated induction of VEGF pathway.	[97]
	Hypoxic cancer cells	Upregulation of miR-135-b, miR-23a, miR-210, miR-494 and Wnt pathway activation.	[101] and enclosed references
	Ovarian cancer	E-cadherin-mediated activation of β-catenin and NFκB (Nuclear Factor kappa-light-chain-enhancer of activated B cells) signaling	[103]
	Cervical squamous cell carcinoma	miR-221-3p-mediated activation of the ERK (Extracellular signal-Regulated Kinase)/AKT pathway	[122]
Immunomodulation	Head and neck squamous cell carcinoma	Receptor–ligand interactions regulating gene expression in T cells	[90]
	Melanoma cells	miR-690 induction of mitochondrial apoptotic pathway in CD4+ T cells	[91]
	Several cancer cells	miRNAs regulation	[92] and enclosed references
	Lung adenocarcinoma, hepatocellular carcinoma, breast carcinoma	Monocyte recruitment and Generation of Tumor Associated Macrofages	[104]
	Hypoxic lung cancer	miR-103a-mediated targeting of PTEN (Phosphatase and tensin homolog) and activation of Tumor Associated Macrofages	[120]
*Chemoresistence and Cancer Stem Cell stimulation*	CAF from colon rectal cancer	Wnt3a induction of WNT signalling activation in CSC (Cancer Stem Cells)	[127]
	CAF from breast cancer	miR-21, miR-378e and mir-143-mediated Cancer Stem Cells maintenance	[113]
	Renal cell carcinoma	lncRNA ARSR-mediated chemoresistance via competitively binding of miR-34/miR-449.	[128]
*Metabolism modulation*	Melanoma cells	miR-155 and miR-210-mediated promotion of glycolysis and inhibition of oxidative phosphorylation.	[88]
	CAF from prostate cancer and from pancreatic cancer	Metabolites inhibiting mitochondrial oxidative phosphorylation and increasing glycolysis.	[89]

## References

[1] Johnstone R.M., Adam M., Hammond J.R., Orr L., Turbide C. (1987). Vesicle formation during reticulocyte maturation. Association of plasma membrane activities with released vesicles (exosomes). J. Biol. Chem..

[2] Pan B.T., Johnstone R.M. (1983). Fate of the transferrin receptor during maturation of sheep reticulocytes in vitro: Selective externalization of the receptor. Cell.

[3] Thierry A.R., El Messaoudi S., Gahan P.B., Anker P., Stroun M. (2016). Origins, structures, and functions of circulating DNA in oncology. Cancer Metastasis Rev..

[4] Mittelbrunn M., Sanchez-Madrid F. (2012). Intercellular communication: Diverse structures for exchange of genetic information. Nat. Rev. Mol. Cell Biol..

[5] Robbins P.D., Morelli A.E. (2014). Regulation of immune responses by extracellular vesicles. Nat. Rev. Immunol..

[6] Bakhti M., Winter C., Simons M. (2011). Inhibition of myelin membrane sheath formation by oligodendrocyte-derived exosome-like vesicles. J. Biol. Chem..

[7] Fruhbeis C., Frohlich D., Kuo W.P., Amphornrat J., Thilemann S., Saab A.S., Kirchhoff F., Mobius W., Goebbels S., Nave K.A. (2013). Neurotransmitter-triggered transfer of exosomes mediates oligodendrocyte-neuron communication. PLoS Biol..

[8] Lopez-Verrilli M.A., Picou F., Court F.A. (2013). Schwann cell-derived exosomes enhance axonal regeneration in the peripheral nervous system. Glia.

[9] Guay C., Regazzi R. (2017). Exosomes as new players in metabolic organ cross-talk. Diabetes Obes. Metab..

[10] Logozzi M., De Milito A., Lugini L., Borghi M., Calabro L., Spada M., Perdicchio M., Marino M.L., Federici C., Iessi E. (2009). High levels of exosomes expressing CD63 and caveolin-1 in plasma of melanoma patients. PLoS ONE.

[11] Peinado H., Aleckovic M., Lavotshkin S., Matei I., Costa-Silva B., Moreno-Bueno G., Hergueta-Redondo M., Williams C., Garcia-Santos G., Ghajar C. (2012). Melanoma exosomes educate bone marrow progenitor cells toward a pro-metastatic phenotype through MET. Nat. Med..

[12] Colombo M., Moita C., van Niel G., Kowal J., Vigneron J., Benaroch P., Manel N., Moita L.F., Thery C., Raposo G. (2013). Analysis of ESCRT functions in exosome biogenesis, composition and secretion highlights the heterogeneity of extracellular vesicles. J. Cell Sci..

[13] Ostrowski M., Carmo N.B., Krumeich S., Fanget I., Raposo G., Savina A., Moita C.F., Schauer K., Hume A.N., Freitas R.P. (2010). Rab27a and Rab27b control different steps of the exosome secretion pathway. Nat. Cell Biol..

[14] Baietti M.F., Zhang Z., Mortier E., Melchior A., Degeest G., Geeraerts A., Ivarsson Y., Depoortere F., Coomans C., Vermeiren E. (2012). Syndecan-syntenin-ALIX regulates the biogenesis of exosomes. Nat. Cell Biol..

[15] Kowal J., Arras G., Colombo M., Jouve M., Morath J.P., Primdal-Bengtson B., Dingli F., Loew D., Tkach M., Thery C. (2016). Proteomic comparison defines novel markers to characterize heterogeneous populations of extracellular vesicle subtypes. Proc. Natl. Acad. Sci. USA.

[16] Willms E., Johansson H.J., Mager I., Lee Y., Blomberg K.E., Sadik M., Alaarg A., Smith C.I., Lehtio J., El Andaloussi S. (2016). Cells release subpopulations of exosomes with distinct molecular and biological properties. Sci. Rep..

[17] Ogawa Y., Miura Y., Harazono A., Kanai-Azuma M., Akimoto Y., Kawakami H., Yamaguchi T., Toda T., Endo T., Tsubuki M. (2011). Proteomic analysis of two types of exosomes in human whole saliva. Biol. Pharm. Bull..

[18] Ogawa Y., Taketomi Y., Murakami M., Tsujimoto M., Yanoshita R. (2013). Small RNA transcriptomes of two types of exosomes in human whole saliva determined by next generation sequencing. Biol. Pharm. Bull..

[19] Lasser C., Shelke G.V., Yeri A., Kim D.K., Crescitelli R., Raimondo S., Sjostrand M., Gho Y.S., van Keuren Jensen K., Lotvall J. (2017). Two distinct extracellular RNA signatures released by a single cell type identified by microarray and next-generation sequencing. RNA Biol..

[20] Bobrie A., Colombo M., Krumeich S., Raposo G., Thery C. (2012). Diverse subpopulations of vesicles secreted by different intracellular mechanisms are present in exosome preparations obtained by differential ultracentrifugation. J. Extracell. Ves..

[21] ExoCarta. http://www.exocarta.org.

[22] Vesiclepedia. http://www.microvesicles.org.

[23] Skog J., Wurdinger T., van Rijn S., Meijer D.H., Gainche L., Sena-Esteves M., Curry W.T., Carter B.S., Krichevsky A.M., Breakefield X.O. (2008). Glioblastoma microvesicles transport RNA and proteins that promote tumour growth and provide diagnostic biomarkers. Nat. Cell Biol..

[24] Conigliaro A., Fontana S., Raimondo S., Alessandro R. (2017). Exosomes: Nanocarriers of Biological Messages. Adv. Exp. Med. Biol..

[25] Nolte-’t Hoen E.N., Buermans H.P., Waasdorp M., Stoorvogel W., Wauben M.H., ’t Hoen P.A. (2012). Deep sequencing of RNA from immune cell-derived vesicles uncovers the selective incorporation of small non-coding RNA biotypes with potential regulatory functions. Nucleic Acids Res..

[26] Mittelbrunn M., Gutierrez-Vazquez C., Villarroya-Beltri C., Gonzalez S., Sanchez-Cabo F., Gonzalez M.A., Bernad A., Sanchez-Madrid F. (2011). Unidirectional transfer of microRNA-loaded exosomes from T cells to antigen-presenting cells. Nat. Commun..

[27] Valadi H., Ekstrom K., Bossios A., Sjostrand M., Lee J.J., Lotvall J.O. (2007). Exosome-mediated transfer of mRNAs and microRNAs is a novel mechanism of genetic exchange between cells. Nat. Cell Biol..

[28] Pegtel D.M., Cosmopoulos K., Thorley-Lawson D.A., van Eijndhoven M.A., Hopmans E.S., Lindenberg J.L., de Gruijl T.D., Wurdinger T., Middeldorp J.M. (2010). Functional delivery of viral miRNAs via exosomes. Proc. Natl. Acad. Sci. USA.

[29] Zhang J., Liu S.C., Luo X.H., Tao G.X., Guan M., Yuan H., Hu D.K. (2016). Exosomal Long Noncoding RNAs are Differentially Expressed in the Cervicovaginal Lavage Samples of Cervical Cancer Patients. J. Clin. Lab. Anal..

[30] Berrondo C., Flax J., Kucherov V., Siebert A., Osinski T., Rosenberg A., Fucile C., Richheimer S., Beckham C.J. (2016). Expression of the Long Non-Coding RNA HOTAIR Correlates with Disease Progression in Bladder Cancer and Is Contained in Bladder Cancer Patient Urinary Exosomes. PLoS ONE.

[31] Conigliaro A., Costa V., Lo Dico A., Saieva L., Buccheri S., Dieli F., Manno M., Raccosta S., Mancone C., Tripodi M. (2015). CD90+ liver cancer cells modulate endothelial cell phenotype through the release of exosomes containing H19 lncRNA. Mol. Cancer.

[32] Santos M.F., Rappa G., Karbanova J., Kurth T., Corbeil D., Lorico A. (2018). VAMP-associated protein-A and oxysterol-binding protein-related protein 3 promote the entry of late endosomes into the nucleoplasmic reticulum. J. Biol. Chem..

[33] Rappa G., Santos M.F., Green T.M., Karbanova J., Hassler J., Bai Y., Barsky S.H., Corbeil D., Lorico A. (2017). Nuclear transport of cancer extracellular vesicle-derived biomaterials through nuclear envelope invagination-associated late endosomes. Oncotarget.

[34] Santangelo L., Giurato G., Cicchini C., Montaldo C., Mancone C., Tarallo R., Battistelli C., Alonzi T., Weisz A., Tripodi M. (2016). The RNA-Binding Protein SYNCRIP Is a Component of the Hepatocyte Exosomal Machinery Controlling MicroRNA Sorting. Cell Rep..

[35] Villarroya-Beltri C., Gutierrez-Vazquez C., Sanchez-Cabo F., Perez-Hernandez D., Vazquez J., Martin-Cofreces N., Martinez-Herrera D.J., Pascual-Montano A., Mittelbrunn M., Sanchez-Madrid F. (2013). Sumoylated hnRNPA2B1 controls the sorting of miRNAs into exosomes through binding to specific motifs. Nat. Commun..

[36] Hobor F., Dallmann A., Ball N.J., Cicchini C., Battistelli C., Ogrodowicz R.W., Christodoulou E., Martin S.R., Castello A., Tripodi M. (2018). A cryptic RNA-binding domain mediates Syncrip recognition and exosomal partitioning of miRNA targets. Nat. Commun..

[37] Parolini I., Federici C., Raggi C., Lugini L., Palleschi S., De Milito A., Coscia C., Iessi E., Logozzi M., Molinari A. (2009). Microenvironmental pH is a key factor for exosome traffic in tumor cells. J. Biol. Chem..

[38] Kucharzewska P., Christianson H.C., Welch J.E., Svensson K.J., Fredlund E., Ringner M., Morgelin M., Bourseau-Guilmain E., Bengzon J., Belting M. (2013). Exosomes reflect the hypoxic status of glioma cells and mediate hypoxia-dependent activation of vascular cells during tumor development. Proc. Natl. Acad. Sci. USA.

[39] French K.C., Antonyak M.A., Cerione R.A. (2017). Extracellular vesicle docking at the cellular port: Extracellular vesicle binding and uptake. Semin. Cell Dev. Biol..

[40] Hoshino A., Costa-Silva B., Shen T.L., Rodrigues G., Hashimoto A., Tesic Mark M., Molina H., Kohsaka S., Di Giannatale A., Ceder S. (2015). Tumour exosome integrins determine organotropic metastasis. Nature.

[41] Chen G., Huang A.C., Zhang W., Zhang G., Wu M., Xu W., Yu Z., Yang J., Wang B., Sun H. (2018). Exosomal PD-L1 contributes to immunosuppression and is associated with anti-PD-1 response. Nature.

[42] Lugini L., Cecchetti S., Huber V., Luciani F., Macchia G., Spadaro F., Paris L., Abalsamo L., Colone M., Molinari A. (2012). Immune surveillance properties of human NK cell-derived exosomes. J. Immunol..

[43] Wei G., Jie Y., Haibo L., Chaoneng W., Dong H., Jianbing Z., Junjie G., Leilei M., Hongtao S., Yunzeng Z. (2017). Dendritic cells derived exosomes migration to spleen and induction of inflammation are regulated by CCR7. Sci. Rep..

[44] Corrado C., Saieva L., Raimondo S., Santoro A., De Leo G., Alessandro R. (2016). Chronic myelogenous leukaemia exosomes modulate bone marrow microenvironment through activation of epidermal growth factor receptor. J. Cell. Mol. Med..

[45] Taverna S., Pucci M., Giallombardo M., Di Bella M.A., Santarpia M., Reclusa P., Gil-Bazo I., Rolfo C., Alessandro R. (2017). Amphiregulin contained in NSCLC-exosomes induces osteoclast differentiation through the activation of EGFR pathway. Sci. Rep..

[46] Raimondo S., Saieva L., Corrado C., Fontana S., Flugy A., Rizzo A., De Leo G., Alessandro R. (2015). Chronic myeloid leukemia-derived exosomes promote tumor growth through an autocrine mechanism. Cell Commun. Signal..

[47] Yu L., Yang F., Jiang L., Chen Y., Wang K., Xu F., Wei Y., Cao X., Wang J., Cai Z. (2013). Exosomes with membrane-associated TGF-beta1 from gene-modified dendritic cells inhibit murine EAE independently of MHC restriction. Eur. J. Immunol..

[48] Sakakura H., Mii S., Hagiwara S., Kato T., Yamamoto N., Hibi H., Takahashi M., Murakumo Y. (2016). CD109 is a component of exosome secreted from cultured cells. Biochem. Biophys. Res. Commun..

[49] Nieto M.A., Huang R.Y., Jackson R.A., Thiery J.P. (2016). Emt: 2016. Cell.

[50] Brabletz T., Jung A., Reu S., Porzner M., Hlubek F., Kunz-Schughart L.A., Knuechel R., Kirchner T. (2001). Variable beta-catenin expression in colorectal cancers indicates tumor progression driven by the tumor environment. Proc. Natl. Acad. Sci. USA.

[51] Dahl U., Sjodin A., Larue L., Radice G.L., Cajander S., Takeichi M., Kemler R., Semb H. (2002). Genetic dissection of cadherin function during nephrogenesis. Mol. Cell. Biol..

[52] Perez-Moreno M.A., Locascio A., Rodrigo I., Dhondt G., Portillo F., Nieto M.A., Cano A. (2001). A new role for E12/E47 in the repression of E-cadherin expression and epithelial-mesenchymal transitions. J. Biol. Chem..

[53] Nieto M.A. (2002). The snail superfamily of zinc-finger transcription factors. Nat. Rev. Mol. Cell Biol..

[54] Yang J., Mani S.A., Donaher J.L., Ramaswamy S., Itzykson R.A., Come C., Savagner P., Gitelman I., Richardson A., Weinberg R.A. (2004). Twist, a master regulator of morphogenesis, plays an essential role in tumor metastasis. Cell.

[55] Rodriguez M., Aladowicz E., Lanfrancone L., Goding C.R. (2008). Tbx3 represses E-cadherin expression and enhances melanoma invasiveness. Cancer Res..

[56] Peinado H., Olmeda D., Cano A. (2007). Snail, Zeb and bHLH factors in tumour progression: An alliance against the epithelial phenotype?. Nat. Rev. Cancer.

[57] Battistelli C., Cicchini C., Santangelo L., Tramontano A., Grassi L., Gonzalez F.J., de Nonno V., Grassi G., Amicone L., Tripodi M. (2017). The Snail repressor recruits EZH2 to specific genomic sites through the enrollment of the lncRNA HOTAIR in epithelial-to-mesenchymal transition. Oncogene.

[58] Battistelli C., Tripodi M., Cicchini C. (2017). Targeting of polycombs to DNA in EMT. Oncotarget.

[59] Yu M., Bardia A., Wittner B.S., Stott S.L., Smas M.E., Ting D.T., Isakoff S.J., Ciciliano J.C., Wells M.N., Shah A.M. (2013). Circulating breast tumor cells exhibit dynamic changes in epithelial and mesenchymal composition. Science.

[60] Huang R.Y., Wong M.K., Tan T.Z., Kuay K.T., Ng A.H., Chung V.Y., Chu Y.S., Matsumura N., Lai H.C., Lee Y.F. (2013). An EMT spectrum defines an anoikis-resistant and spheroidogenic intermediate mesenchymal state that is sensitive to e-cadherin restoration by a src-kinase inhibitor, saracatinib (AZD0530). Cell Death Dis..

[61] Schliekelman M.J., Taguchi A., Zhu J., Dai X., Rodriguez J., Celiktas M., Zhang Q., Chin A., Wong C.H., Wang H. (2015). Molecular portraits of epithelial, mesenchymal, and hybrid States in lung adenocarcinoma and their relevance to survival. Cancer Res..

[62] Pastushenko I., Brisebarre A., Sifrim A., Fioramonti M., Revenco T., Boumahdi S., Van Keymeulen A., Brown D., Moers V., Lemaire S. (2018). Identification of the tumour transition states occurring during EMT. Nature.

[63] Ruscetti M., Quach B., Dadashian E.L., Mulholland D.J., Wu H. (2015). Tracking and Functional Characterization of Epithelial-Mesenchymal Transition and Mesenchymal Tumor Cells during Prostate Cancer Metastasis. Cancer Res..

[64] Yamashita N., Tokunaga E., Iimori M., Inoue Y., Tanaka K., Kitao H., Saeki H., Oki E., Maehara Y. (2018). Epithelial Paradox: Clinical Significance of Coexpression of E-cadherin and Vimentin With Regard to Invasion and Metastasis of Breast Cancer. Clin. Breast Cancer.

[65] Conigliaro A., Amicone L., Costa V., De Santis Puzzonia M., Mancone C., Sacchetti B., Cicchini C., Garibaldi F., Brenner D.A., Kisseleva T. (2013). Evidence for a common progenitor of epithelial and mesenchymal components of the liver. Cell Death Differ..

[66] Garibaldi F., Cicchini C., Conigliaro A., Santangelo L., Cozzolino A.M., Grassi G., Marchetti A., Tripodi M., Amicone L. (2012). An epistatic mini-circuitry between the transcription factors Snail and HNF4alpha controls liver stem cell and hepatocyte features exhorting opposite regulation on stemness-inhibiting microRNAs. Cell Death Differ..

[67] Diaz-Lopez A., Moreno-Bueno G., Cano A. (2014). Role of microRNA in epithelial to mesenchymal transition and metastasis and clinical perspectives. Cancer Manag. Res..

[68] Costa V., Lo Dico A., Rizzo A., Rajata F., Tripodi M., Alessandro R., Conigliaro A. (2017). MiR-675-5p supports hypoxia induced epithelial to mesenchymal transition in colon cancer cells. Oncotarget.

[69] Jolly M.K., Tripathi S.C., Jia D., Mooney S.M., Celiktas M., Hanash S.M., Mani S.A., Pienta K.J., Ben-Jacob E., Levine H. (2016). Stability of the hybrid epithelial/mesenchymal phenotype. Oncotarget.

[70] Lu M., Jolly M.K., Levine H., Onuchic J.N., Ben-Jacob E. (2013). MicroRNA-based regulation of epithelial-hybrid-mesenchymal fate determination. Proc. Natl. Acad. Sci. USA.

[71] Tian X.J., Zhang H., Xing J. (2013). Coupled reversible and irreversible bistable switches underlying TGFbeta-induced epithelial to mesenchymal transition. Biophys. J..

[72] Kalluri R. (2016). The biology and function of exosomes in cancer. J. Clin. Investig..

[73] Green T.M., Alpaugh M.L., Barsky S.H., Rappa G., Lorico A. (2015). Breast Cancer-Derived Extracellular Vesicles: Characterization and Contribution to the Metastatic Phenotype. Biomed. Res. Int..

[74] Cesi G., Walbrecq G., Margue C., Kreis S. (2016). Transferring intercellular signals and traits between cancer cells: Extracellular vesicles as “homing pigeons”. Cell Commun. Signal..

[75] You Y., Shan Y., Chen J., Yue H., You B., Shi S., Li X., Cao X. (2015). Matrix metalloproteinase 13-containing exosomes promote nasopharyngeal carcinoma metastasis. Cancer Sci..

[76] Zomer A., Maynard C., Verweij F.J., Kamermans A., Schafer R., Beerling E., Schiffelers R.M., de Wit E., Berenguer J., Ellenbroek S.I. (2015). In Vivo imaging reveals extracellular vesicle-mediated phenocopying of metastatic behavior. Cell.

[77] Tang M.K., Wong A.S. (2015). Exosomes: Emerging biomarkers and targets for ovarian cancer. Cancer Lett..

[78] Aga M., Bentz G.L., Raffa S., Torrisi M.R., Kondo S., Wakisaka N., Yoshizaki T., Pagano J.S., Shackelford J. (2014). Exosomal HIF1alpha supports invasive potential of nasopharyngeal carcinoma-associated LMP1-positive exosomes. Oncogene.

[79] Franzen C.A., Blackwell R.H., Todorovic V., Greco K.A., Foreman K.E., Flanigan R.C., Kuo P.C., Gupta G.N. (2015). Urothelial cells undergo epithelial-to-mesenchymal transition after exposure to muscle invasive bladder cancer exosomes. Oncogenesis.

[80] Jeppesen D.K., Nawrocki A., Jensen S.G., Thorsen K., Whitehead B., Howard K.A., Dyrskjot L., Orntoft T.F., Larsen M.R., Ostenfeld M.S. (2014). Quantitative proteomics of fractionated membrane and lumen exosome proteins from isogenic metastatic and nonmetastatic bladder cancer cells reveal differential expression of EMT factors. Proteomics.

[81] Wang J., Guan X., Zhang Y., Ge S., Zhang L., Li H., Wang X., Liu R., Ning T., Deng T. (2018). Exosomal miR-27a Derived from Gastric Cancer Cells Regulates the Transformation of Fibroblasts into Cancer-Associated Fibroblasts. Cell. Physiol. Biochem..

[82] Wang J., Yang X., Li R., Wang L., Gu Y., Zhao Y., Huang K.H., Cheng T., Yuan Y., Gao S. (2018). Long non-coding RNA MYU promotes prostate cancer proliferation by mediating the miR-184/c-Myc axis. Oncol. Rep..

[83] Kalluri R., Zeisberg M. (2006). Fibroblasts in cancer. Nat. Rev. Cancer.

[84] Ringuette Goulet C., Bernard G., Tremblay S., Chabaud S., Bolduc S., Pouliot F. (2018). Exosomes Induce Fibroblast Differentiation into Cancer-Associated Fibroblasts through TGFbeta Signaling. Mol. Cancer Res..

[85] Principe S., Mejia-Guerrero S., Ignatchenko V., Sinha A., Ignatchenko A., Shi W., Pereira K., Su S., Huang S.H., O’Sullivan B. (2018). Proteomic Analysis of Cancer-Associated Fibroblasts Reveals a Paracrine Role for MFAP5 in Human Oral Tongue Squamous Cell. Carcinoma. J. Proteome Res..

[86] Herrera M., Llorens C., Rodriguez M., Herrera A., Ramos R., Gil B., Candia A., Larriba M.J., Garre P., Earl J. (2018). Differential distribution and enrichment of non-coding RNAs in exosomes from normal and Cancer-associated fibroblasts in colorectal cancer. Mol. Cancer.

[87] Zhang Z., Li X., Sun W., Yue S., Yang J., Li J., Ma B., Wang J., Yang X., Pu M. (2017). Loss of exosomal miR-320a from cancer-associated fibroblasts contributes to HCC proliferation and metastasis. Cancer Lett..

[88] La Shu S., Yang Y., Allen C.L., Maguire O., Minderman H., Sen A., Ciesielski M.J., Collins K.A., Bush P.J., Singh P. (2018). Metabolic reprogramming of stromal fibroblasts by melanoma exosome microRNA favours a pre-metastatic microenvironment. Sci. Rep..

[89] Zhao H., Yang L., Baddour J., Achreja A., Bernard V., Moss T., Marini J.C., Tudawe T., Seviour E.G. (2016). Tumor microenvironment derived exosomes pleiotropically modulate cancer cell metabolism. Elife.

[90] Muller L., Mitsuhashi M., Simms P., Gooding W.E., Whiteside T.L. (2016). Tumor-derived exosomes regulate expression of immune function-related genes in human T cell subsets. Sci. Rep..

[91] Zhou J., Yang Y., Wang W., Zhang Y., Chen Z., Hao C., Zhang J. (2018). Melanoma-released exosomes directly activate the mitochondrial apoptotic pathway of CD4(+) T cells through their microRNA cargo. Exp. Cell Res..

[92] Hirschberger S., Hinske L.C., Kreth S. (2018). MiRNAs: Dynamic regulators of immune cell functions in inflammation and cancer. Cancer Lett..

[93] Clayton A., Al-Taei S., Webber J., Mason M.D., Tabi Z. (2011). Cancer exosomes express CD39 and CD73, which suppress T cells through adenosine production. J. Immunol..

[94] Costa-Silva B., Aiello N.M., Ocean A.J., Singh S., Zhang H., Thakur B.K., Becker A., Hoshino A., Mark M.T., Molina H. (2015). Pancreatic cancer exosomes initiate pre-metastatic niche formation in the liver. Nat. Cell Biol..

[95] Zhou W., Fong M.Y., Min Y., Somlo G., Liu L., Palomares M.R., Yu Y., Chow A., O’Connor S.T., Chin A.R. (2014). Cancer-secreted miR-105 destroys vascular endothelial barriers to promote metastasis. Cancer Cell.

[96] Grange C., Tapparo M., Collino F., Vitillo L., Damasco C., Deregibus M.C., Tetta C., Bussolati B., Camussi G. (2011). Microvesicles released from human renal cancer stem cells stimulate angiogenesis and formation of lung premetastatic niche. Cancer Res..

[97] Sun X., Ma X., Wang J., Zhao Y., Wang Y., Bihl J.C., Chen Y., Jiang C. (2017). Glioma stem cells-derived exosomes promote the angiogenic ability of endothelial cells through miR-21/VEGF signal. Oncotarget.

[98] Takasugi M., Okada R., Takahashi A., Virya Chen D., Watanabe S., Hara E. (2017). Small extracellular vesicles secreted from senescent cells promote cancer cell proliferation through EphA2. Nat. Commun..

[99] Petrova V., Annicchiarico-Petruzzelli M., Melino G., Amelio I. (2018). The hypoxic tumour microenvironment. Oncogenesis.

[100] Zhang W., Zhou X., Yao Q., Liu Y., Zhang H., Dong Z. (2017). HIF-1-mediated production of exosomes during hypoxia is protective in renal tubular cells. Am. J. Physiol. Ren. Physiol..

[101] Shao C., Yang F., Miao S., Liu W., Wang C., Shu Y., Shen H. (2018). Role of hypoxia-induced exosomes in tumor biology. Mol. Cancer.

[102] Dey N., De P., Brian L.J. (2015). Evading anti-angiogenic therapy: Resistance to anti-angiogenic therapy in solid tumors. Am. J. Transl. Res..

[103] Tang M.K.S., Yue P.Y.K., Ip P.P., Huang R.L., Lai H.C., Cheung A.N.Y., Tse K.Y., Ngan H.Y.S., Wong A.S.T. (2018). Soluble E-cadherin promotes tumor angiogenesis and localizes to exosome surface. Nat. Commun..

[104] Song X., Ding Y., Liu G., Yang X., Zhao R., Zhang Y., Zhao X., Anderson G.J., Nie G. (2016). Cancer Cell-derived Exosomes Induce Mitogen-activated Protein Kinase-dependent Monocyte Survival by Transport. of Functional Receptor Tyrosine Kinases. J. Biol. Chem..

[105] Rahman M.A., Barger J.F., Lovat F., Gao M., Otterson G.A., Nana-Sinkam P. (2016). Lung cancer exosomes as drivers of epithelial mesenchymal transition. Oncotarget.

[106] Chen L., Guo P., He Y., Chen Z., Chen L., Luo Y., Qi L., Liu Y., Wu Q., Cui Y. (2018). HCC-derived exosomes elicit HCC progression and recurrence by epithelial-mesenchymal transition through MAPK/ERK signalling pathway. Cell Death Dis..

[107] Higginbotham J.N., Demory Beckler M., Gephart J.D., Franklin J.L., Bogatcheva G., Kremers G.J., Piston D.W., Ayers G.D., McConnell R.E., Tyska M.J. (2011). Amphiregulin exosomes increase cancer cell invasion. Curr. Biol..

[108] Harris D.A., Patel S.H., Gucek M., Hendrix A., Westbroek W., Taraska J.W. (2015). Exosomes released from breast cancer carcinomas stimulate cell movement. PLoS ONE.

[109] Sung B.H., Ketova T., Hoshino D., Zijlstra A., Weaver A.M. (2015). Directional cell movement through tissues is controlled by exosome secretion. Nat. Commun..

[110] Schillaci O., Fontana S., Monteleone F., Taverna S., Di Bella M.A., Di Vizio D., Alessandro R. (2017). Exosomes from metastatic cancer cells transfer amoeboid phenotype to non-metastatic cells and increase endothelial permeability: Their emerging role in tumor heterogeneity. Sci. Rep..

[111] Dong L., Lin W., Qi P., Xu M.D., Wu X., Ni S., Huang D., Weng W.W., Tan C., Sheng W. (2016). Circulating Long RNAs in Serum Extracellular Vesicles: Their Characterization and Potential Application as Biomarkers for Diagnosis of Colorectal Cancer. Cancer Epidemiol. Biomarkers Prev..

[112] Battistelli C., Sabarese G., Santangelo L., Montaldo C., Gonzalez F.J., Tripodi M., Cicchini C. (2018). The lncRNA HOTAIR transcription is controlled by HNF4alpha-induced chromatin topology modulation. Cell Death Differ..

[113] Amicone L., Citarella F., Cicchini C. (2015). Epigenetic regulation in hepatocellular carcinoma requires long noncoding RNAs. Biomed. Res. Int..

[114] Luga V., Zhang L., Viloria-Petit A.M., Ogunjimi A.A., Inanlou M.R., Chiu E., Buchanan M., Hosein A.N., Basik M., Wrana J.L. (2012). Exosomes mediate stromal mobilization of autocrine Wnt-PCP signaling in breast cancer cell migration. Cell.

[115] Donnarumma E., Fiore D., Nappa M., Roscigno G., Adamo A., Iaboni M., Russo V., Affinito A., Puoti I., Quintavalle C. (2017). Cancer-associated fibroblasts release exosomal microRNAs that dictate an aggressive phenotype in breast cancer. Oncotarget.

[116] Li W., Zhang X., Wang J., Li M., Cao C., Tan J., Ma D., Gao Q. (2017). TGFbeta1 in fibroblasts-derived exosomes promotes epithelial-mesenchymal transition of ovarian cancer cells. Oncotarget.

[117] Zhao X., Wu X., Qian M., Song Y., Wu D., Zhang W. (2018). Knockdown of TGF-beta1 expression in human umbilical cord mesenchymal stem cells reverts their exosome-mediated EMT promoting effect on lung cancer cells. Cancer Lett..

[118] Ramteke A., Ting H., Agarwal C., Mateen S., Somasagara R., Hussain A., Graner M., Frederick B., Agarwal R., Deep G. (2015). Exosomes secreted under hypoxia enhance invasiveness and stemness of prostate cancer cells by targeting adherens junction molecules. Mol. Carcinog..

[119] Xue M., Chen W., Xiang A., Wang R., Chen H., Pan J., Pang H., An H., Wang X., Hou H. (2017). Hypoxic exosomes facilitate bladder tumor growth and development through transferring long non-coding RNA-UCA1. Mol. Cancer.

[120] Takahashi K., Yan I.K., Haga H., Patel T. (2014). Modulation of hypoxia-signaling pathways by extracellular linc-RoR. J. Cell Sci..

[121] Hsu Y.L., Hung J.Y., Chang W.A., Jian S.F., Lin Y.S., Pan Y.C., Wu C.Y., Kuo P.L. (2018). Hypoxic Lung-Cancer-Derived Extracellular Vesicle MicroRNA-103a Increases the Oncogenic Effects of Macrophages by Targeting PTEN. Mol. Ther..

[122] Zhou C.F., Ma J., Huang L., Yi H.Y., Zhang Y.M., Wu X.G., Yan R.M., Liang L., Zhong M., Yu Y.H. (2018). Cervical squamous cell carcinoma-secreted exosomal miR-221-3p promotes lymphangiogenesis and lymphatic metastasis by targeting VASH1. Oncogene.

[123] Karaosmanoglu O., Banerjee S., Sivas H. (2018). Identification of biomarkers associated with partial epithelial to mesenchymal transition in the secretome of slug over-expressing hepatocellular carcinoma cells. Cell. Oncol..

[124] Grosse-Wilde A., Fouquier d’Herouel A., McIntosh E., Ertaylan G., Skupin A., Kuestner R.E., del Sol A., Walters K.A., Huang S. (2015). Stemness of the hybrid Epithelial/Mesenchymal State in Breast Cancer and Its Association with Poor Survival. PLoS ONE.

[125] Richards K.E., Zeleniak A.E., Fishel M.L., Wu J., Littlepage L.E., Hill R. (2017). Cancer-associated fibroblast exosomes regulate survival and proliferation of pancreatic cancer cells. Oncogene.

[126] Singh R., Pochampally R., Watabe K., Lu Z., Mo Y.Y. (2014). Exosome-mediated transfer of miR-10b promotes cell invasion in breast cancer. Mol. Cancer.

[127] Hu Y., Yan C., Mu L., Huang K., Li X., Tao D., Wu Y., Qin J. (2015). Fibroblast-Derived Exosomes Contribute to Chemoresistance through Priming Cancer Stem Cells in Colorectal Cancer. PLoS ONE.

[128] Qu L., Ding J., Chen C., Wu Z.J., Liu B., Gao Y., Chen W., Liu F., Sun W., Li X.F. (2016). Exosome-Transmitted lncARSR Promotes Sunitinib Resistance in Renal Cancer by Acting as a Competing Endogenous RNA. Cancer Cell.

[129] Bolukbasi M.F., Mizrak A., Ozdener G.B., Madlener S., Strobel T., Erkan E.P., Fan J.B., Breakefield X.O., Saydam O. (2012). miR-1289 and “Zipcode”-like Sequence Enrich. mRNAs in Microvesicles. Mol. Ther. Nucleic Acids.

[130] Hirsova P., Ibrahim S.H., Krishnan A., Verma V.K., Bronk S.F., Werneburg N.W., Charlton M.R., Shah V.H., Malhi H., Gores G.J. (2016). Lipid-Induced Signaling Causes Release of Inflammatory Extracellular Vesicles From Hepatocytes. Gastroenterology.

[131] Savina A., Furlan M., Vidal M., Colombo M.I. (2003). Exosome release is regulated by a calcium-dependent mechanism in K562 cells. J. Biol. Chem..

[132] Kim M.S., Haney M.J., Zhao Y., Yuan D., Deygen I., Klyachko N.L., Kabanov A.V., Batrakova E.V. (2018). Engineering macrophage-derived exosomes for targeted paclitaxel delivery to pulmonary metastases: In vitro and in vivo evaluations. Nanomedicine.

